# On Diseases of the Lungs and Pleuræ, Including Tuberculosis and Mediastinal Growths

**Published:** 1911-12

**Authors:** 


**On Diseases of the Lungs and Pleuræ, including Tuberculosis and Mediastinal Growths.** By Sir R. DOUGLAS POWELL, Bart., K.C.V.O., M.D., and P. HORTON-SMITH HARTLEY, M.V.O., M.A., M.D. Fifth Edition. Pp. xxviii., 712. London: H. K. Lewis. 1911.

The fifth edition of this most useful work contains many changes, the text having been largely rewritten, many new illustrations being added and the references brought up to date. The book is a model of clear, concise and helpful teaching. During the eighteen years that have elapsed since the publication of the fourth edition there have been great changes in medical opinion with regard to the ætiology and treatment of pulmonary tuberculosis, and the authors have devoted much space to the consideration of these points. Recently the results of the sanatorium treatment of phthisis have been somewhat adversely criticised, and there has been a reaction in favour of the tuberculin treatment. The medical man seeking information on these complex questions cannot do better than read the section devoted to the result of sanatorium treatment and the chapter on the specific treatment of pulmonary tuberculosis. The matter is summed up thoroughly and impartially, and in view of approaching legislation providing for State sanatoria, it should be carefully studied by all members of the profession.

